# Operating theatre related syncope in medical students: a cross sectional study

**DOI:** 10.1186/1472-6920-9-14

**Published:** 2009-03-10

**Authors:** AAB Jamjoom, A Nikkar-Esfahani, JEF Fitzgerald

**Affiliations:** 1 University of Nottingham Medical School, Queen's Medical Centre, Nottingham, NG7 2UH, UK; 2 Department of Gastrointestinal Surgery, Nottingham University Hospitals NHS Trust, Nottingham, NG7 2UH, UK; 3 Medical Education Unit, University of Nottingham Medical School, Nottingham University Hospital, Nottingham, NG7 2UH, UK

## Abstract

**Background:**

Observing surgical procedures is a beneficial educational experience for medical students during their surgical placements. Anecdotal evidence suggests that operating theatre related syncope may have detrimental effects on students' views of this. Our study examines the frequency and causes of such syncope, together with effects on career intentions, and practical steps to avoid its occurrence.

**Methods:**

All penultimate and final year students at a large UK medical school were surveyed using the University IT system supplemented by personal approach. A 20-item anonymous questionnaire was distributed and results were analysed using the Statistical Package for Social Sciences, version 15.0 (Chicago, Illinois, USA).

**Results:**

Of the 630 clinical students surveyed, 77 responded with details of at least one near or actual operating theatre syncope (12%). A statistically significant gender difference existed for syncopal/near-syncopal episodes (male 12%; female 88%), p < 0.05. Twenty-two percent of those affected were graduate entry medical course students with the remaining 78% undergraduate. Mean age was 23-years (range 20 – 45). Of the 77 reactors, 44 (57%) reported an intention to pursue a surgical career. Of this group, 7 (9%) reported being discouraged by syncopal episodes in the operating theatre. The most prevalent contributory factors were reported as hot temperature (n = 61, 79%), prolonged standing (n = 56, 73%), wearing a surgical mask (n = 36, 47%) and the smell of diathermy (n = 18, 23%). The most frequently reported measures that students found helpful in reducing the occurrence of syncopal episodes were eating and drinking prior to attending theatre (n = 47, 61%), and moving their legs whilst standing (n = 14, 18%).

**Conclusion:**

Our study shows that operating theatre related syncope among medical students is common, and we establish useful risk factors and practical steps that have been used to prevent its occurrence. Our study also highlights the detrimental effect of this on the career intentions of medical students interested in surgery. Based on these findings, we recommend that dedicated time should be set aside in surgical teaching to address this issue prior to students attending the operating theatre.

## Background

Core knowledge, skills and competencies for practice as a junior doctor are developed at medical school. Students gain experience through clinical placements in a range of specialities. Surgical clerkships are a key constituent of this, allowing students to understand surgical disease in context through working with surgeons and their patients. Surgical attachments are also important in enabling students to acquire additional skills, such as assisting in the operating theatre or gaining proficiency at basic procedures such as suturing.

Observing operative procedures enables medical students to develop their understanding of the patient journey through surgery, the care pathways involved and the differing roles of theatre staff. It is an opportunity to learn about teamwork, risk assessment and theatre etiquette. Importantly, students also observe treatment of the underlying condition, learning relevant pathology, anatomy, operations, and the risks associated with these. As such, the operating theatre plays an essential role in delivering numerous aspects of the modern medical school curriculum [1].

Surgical diseases do not respect artificial boundaries between different medical specialities, and it is important for all students to gain a working knowledge of these, regardless of ultimate career intentions. However, in comparison to other areas of medical education, there is a relative paucity of research surrounding student time spent observing surgery.

Syncopal episodes, and fear of these, are one factor potentially influencing medical student attendance in the operating theatre. Such episodes can occur amongst students of any healthcare discipline, but appear to be infrequent amongst qualified theatre staff. These episodes occur as a result of falling blood pressure, precipitated by various environmental or emotional factors [2]. The physiological response protects the brain against potential damage from low perfusion by precipitating collapse and reducing the effect of gravity on the circulation.

Anecdotally, such fainting is a frequent occurrence amongst medical students. However, appropriate warnings or educational advice regarding this are rarely given, and experiences on parallel clerkships have been shown to differ greatly [3]. Indeed, the topic is a 'rite of passage' for many, surrounded by myth, and regarded in a humorous, disparaging or unsympathetic light by many senior theatre staff.

It is already known that such episodes can be a significant source of anxiety for students prior to and during their surgical attachments [4]. Medical student fainting may be harmful to the student, and also to the patient if it interferes with their operation. It may adversely affect learning, discouraging operating theatre attendance or stigmatising those experiencing such an episode. Lack of educational support or preparation within the curriculum may exacerbate this, and the negative response of theatre staff may discourage those interested in surgery. Health and safety issues are also raised, as the absence of adequate educational preparation may in future become grounds for complaint against a hospital or medical school should harm occur to a student or patient.

No previous research has sought to fully quantify the problem of operating theatre related syncope amongst medical students. This study sets out to investigate prevalence, associated factors and gender-specific differences among the clinical students of a large UK medical school. We seek to establish the effects of this on students' perception of a surgical career, and report practical techniques identified by students themselves for preventing syncopal episodes.

## Methods

### Subjects and settings

Students in the final and penultimate year of study at the University of Nottingham medical school were invited to take part in this study in December 2007. There were a total of 630 students in the fourth (penultimate) and fifth (final) year of the medical school course, comprising of 356 (57%) female and 274 (43%) male students. Graduate-entry medical students totalled 165 of these, with the remaining 465 pursuing the standard undergraduate course. Within the medical school curriculum students are taught about the underlying physiology of orthostatic blood pressure control, syncope and the vasovagal response during the first year of their degree. Initial operating theatre attendance begins during their third year as part of their clinical placements.

### Questionnaire

A questionnaire was designed to explore students' views and experience of operating theatre related syncope. The questionnaire allowed for free text and multiple-choice responses addressing the following 6 areas:

1) Demographic details including age and gender.

2) Frequency and associated symptoms of the syncopal episodes.

3) Operation type, medical student role in theatre and any effects on the operation.

4) Effects of syncopal episodes on career choice.

5) Predisposing factors leading to syncopal episodes.

6) Measures taken to prevent syncopal episodes and the effectiveness of these.

The questionnaire was initially piloted by 15 students who were known to have experienced syncopal episodes in the operating theatre. This revealed ambiguities which were amended.

The questionnaire was distributed using the University of Nottingham email system supplemented by personal approach. All fourth and fifth year medical students at the University of Nottingham were invited to participate. Reminder emails were subsequently sent to maximise response rate.

This study was undertaken by the University of Nottingham Medical School Medical Education Unit as part of an audit of medical student experience of surgical teaching attachments and teaching provision. The authors gave due consideration to the ethical dimensions of this anonymous questionnaire survey and no concerns were identified. The questionnaire was optional and completion was taken as consent to participate.

### Analysis of results

Analysis of results was carried out using the Statistical Package for Social Sciences, version 15.0 (Chicago, Illinois, USA). P values were calculated using chi-squared test.

## Results

Out of the 630 students invited to participate, a total of 77 (12%) reported details of near or actual operating theatre related syncope, with no response from the remainder. Twenty-five (4%) of these students reported actual loss of consciousness. The mean age of reactors was 23-years-old (range 20 – 45). The standard undergraduate course yielded 60 responses (78%) with 17 (22%) from the smaller graduate entry medicine course. Sixty-eight reactors were female (88%), with 9 (12%) males. The gender difference was statistically significant. A summary of the demographics by course is given in Table 1.

**Table 1:** Summary of Reactants Characteristics

**Reactants**	**Proportion Percentage (n)**	**Male Percentage (n)**	**Female Percentage (n)**	**Median age (range)**
**Undergraduate Course**	78 (n = 60)	7 (n = 5)	71 (n = 55)	21 (20–48)

**Post Graduate Course**	22 (n = 17)	5 (n = 4)	17 (n = 13)	27 (23–48)

**Overall**	100 (n = 77)	12 (n = 9)	88 (n = 68)	-

### Impact of operating theatre related syncope on career choice

Of the 77 students who replied with at least one near or actual syncopal episode 44 (57%) reported an intention to pursue a surgical career. Of this group, 7 (9%) reported being put off due to syncopal episodes in the operating theatre. Of 33 (43%) reactors who reported an interest in pursuing non-surgical careers, 8 (10%) reported that experiencing syncopal episodes had further discouraged them from a surgical career.

### Settings and immediate consequences

Reactors were asked to report the speciality and type of operation in which they experienced syncopal episodes. Gynaecological operations were cited in 22 cases (29%), followed by 12 (16%) citations for colorectal and 12 (16%) vascular surgery. Procedures involving laparotomy accounted for 27 (35%) of the reported episodes, compared to 12 (16%) in laparoscopy procedures.

Just over half the syncopal episodes occurred when the student was scrubbed (52%). Thirty-six episodes (47%) occurred when the student was assisting in the operation.

There was no report of injury, although the operation was affected in 7 (9%) of cases. This most commonly involved a change of assistant causing delays to surgery.

### Factors contributing to operating theatre related syncope

Forty-eight percent of students who experienced syncopal episodes in the operating theatre had suffered from syncope before the start of their clinical attachments. Students were also asked to comment on factors they thought predisposed them to operating theatre related syncope. Sixty one (79%) cited hot temperature and 56 (73%) cited length of time standing. Contributing factors are summarised in Table 2.

**Table 2:** Summary of most prevalent self-rated contributory factors

**Self-rated factor**	**Percentage of reactants (n)**
**Hot temperature**	79 (n = 61)

**Length of time standing**	73 (n = 56)

**Surgical mask**	47 (n = 36)

**Smell of diathermy**	23 (n = 18)

**Menstruation**	17 (n = 13)

### Preventative measures are effective

Reactors were invited to comment on preventative measures they had taken (if any) to prevent syncopal episodes reoccurring. Of those responding 65 (87%) reported having taken preventative measures to avoid further episodes. No prompting or suggestions of possible measures were provided. The most frequently cited preventative measures are summarised in Table 3.

**Table 3:** Summary of actions taken by 77 medical students to prevent a second occurrence

**Action taken**	**Percentage of reactants (%)**
**Eating and drinking prior to operation**	61 (n = 27)

**Moving legs during the operation**	18 (n = 14)

**Sitting down**	18 (n = 14)

**Taking breaks from assisting**	12 (n = 9)

**Increased attendance to the operating theatre**	10 (n = 8)

Of the students who had taken preventative measures, 60 (78%) commented on their effectiveness. Of these 38 (49%) found these measures effective and 6 (7%) stated that such measures made no difference in the frequency of syncopal episodes in the operating theatre.

## Discussion

This study highlights the prevalence of operating theatre related syncope among medical students, and demonstrates the potentially damaging affect of this phenomenon in discouraging students from pursuing a surgical career.

Attending operations is an essential part of surgical placements at medical schools. In addition, observing and assisting in surgical procedures has been shown to improve students understanding of surgical pathologies and clinical anatomy [5].

Adapting to the new environment of the operating theatre has been shown to be one of several challenges facing medical students at the start of their surgical clerkships [6]. One major element is the anxiety surrounding the potential for syncopal episodes in the operating theatre [4].

The UK Medical Research Group identified that 44.9% of its' respondents believe their ultimate choice of career had been influenced by their undergraduate experience of a speciality [7]. This undergraduate experience has gained even more significance in the United Kingdom following the introduction of the Modernising Medical Careers initiative. Career choices must be made earlier, with reduced flexibility and time in training further reducing the experience that graduates obtain in a given field prior to specialising. This study indicates that operating theatre related syncope can affect students' choice of career and discourage students from pursuing surgery. This becomes worrisome when one considers the gradual decline in numbers of junior doctors pursuing a career in general surgery [8].

This study highlights the impact student experience of the operating theatre can have on their interest in a surgical career. Sixteen percent of students who were interested in pursuing this indicated that they were put off as a result of their syncopal episodes. This may be due to the anxiety that syncopal episodes can cause. Frequent episodes of operating theatre syncope can also reduce the confidence of a medical student in his or her ability to become a surgeon.

Concerns have previously been raised about the potential safety hazard of medical students in theatre, given their reduced experience and knowledge of procedures and aseptic techniques [9]. A lack of appropriate student preparation in their course prior to attending the operating theatre no doubt increased these risks.

Our study shows a high prevalence of operating theatre related syncope among medical students with over 1-in-10 clinical students reporting at least one episode of near or actual syncope. Although two previous studies have reported syncope rates amongst medical students [10,11], we are not aware of any studies that have specifically investigated this in the operating theatre setting. Ganzeboom et al demonstrated that syncope in young adults is common, with 1-in-3 expected to have had at least one episode in their life time [10]. Serletis et al found that gender and family history were important predictors of syncope in medical students, and noted a similar syncopal event rate of 32% [11]

This study identifies hot temperature and prolonged standing to be the major predisposing factors for syncopal episodes, which is consistent with the previous work in this area [10]. Our study also demonstrates that being scrubbed does not predispose students to a higher risk of syncope as 48% of episodes occurred when students were not scrubbed. Furthermore, 16% of episodes occurred during laparoscopic surgery, suggesting that sight of the operation remains a strong contributory factor even when the associated stimulations of open surgery are removed.

None of the reactants in this study identified needle or blood phobias as a cause of their syncopal attacks. This has been demonstrated as a major cause of vasovagal syncope in members of the public [12]. This difference may lie in the fact that those with severe blood and needle phobias may be deterred from entering medical careers.

A statistically significant difference in the incidence of syncope exists between genders in this study. This is consistent with the work carried out by Fu et al and Ganzeboom et al which demonstrated a higher rate of syncope among women in comparison to men. The aetiology of this remains unclear and various mechanisms such as smaller hearts, lower vascular resistance and the hypo-adrenergic response of women have been proposed [13]. The increased willingness of female medical students to own up to their syncopal episodes can also contribute to the increased reported prevalence of such episodes [10,13].

The reported variation in frequency of episodes between different surgical specialities was not statistically significant. Irrespective of this, these figures would reflect the different operating theatre opportunities available in different medical school placements.

Previous studies have described how prior teaching and preparation for theatres such as theatre tours and information on theatre etiquette can improve theatre based learning [14,15]. The high prevalence of syncopal episodes in our study indicates a greater need for students to be informed of potential dangers, contributory factors and preventative measures as part of such induction programmes. Wieling et al highlighted several physical manoeuvres such as leg crossing, muscle tensing and squatting that are effective at combating orthostatic intolerance [16]. Instruction on such manoeuvres or direction towards syncope advice groups may be of use for students commencing their surgical attachments [17]. The results of this study indicate that self-initiated preventative measures, such as eating prior to attending theatre were considered successful by students.

Universities should reflect on the trend towards rising proportions of female medical students, given the importance of undergraduates gaining experience in surgical specialities. The higher prevalence of syncope among females should not be allowed to discourage these students from gaining experience in this area or pursuing a surgical career.

As with any questionnaire survey, a degree of responder bias may affect these results. It is therefore important to note that any under-reporting in this study strengthens the results by further increasing the prevalence of operating theatre related syncope. Given the findings from more generalised medical student studies of a higher syncopal event rate [10,11], it is likely a degree of under-reporting has indeed occurred. However, students who have taken part in this study may not have had equal exposure to operating theatres as attendance may be dependent on the student's underlying interest in surgery.

Finally this study reports the experience of one UK medical school. Although low rates of theatre instruction and a lack of student information have been previously reported at other UK medical schools [14,15] it is unclear as to what degree these results may be extrapolated elsewhere.

We hope that research groups will seek to confirm these findings in other surgical and educational settings. Further studies are then required to devise and assess the effectiveness of different educational measures in addressing operating theatre related syncope. One medical school, recognising the limited preparation students receive for the operating theatre, has recently developed an instructional DVD and learning guide into which such material might be incorporated [18]. It is hoped that this work will increase medical students' educational opportunities in the operating theatre, whilst reducing anxiety and stimulating students to pursue a surgical career.

## Conclusion

Our study indicates that operating theatre related syncope among medical students is common, and highlights the discouraging effect of syncopal episodes on students interested in pursuing a surgical career. Preventative measures are effective and we establish useful risk factors and practical steps to avoid its occurrence. Our results suggest that dedicated time should be set aside in surgical teaching to address this issue prior to trainees attending the operating theatre.

## Competing interests

The authors declare that they have no competing interests.

## Authors' contributions

AABJ contributed to the conception and design of this project, collected data and drafted the manuscript. ANE conceived the original project idea, contributed to the design of the project, collected data and drafted the manuscript. JEFF contributed to the conception and design of this project, coordinated data collection, drafted the paper and revised the final manuscript for submission. All authors have read and approved the final manuscript.

## Pre-publication history

The pre-publication history for this paper can be accessed here:


